# Regulation of hepatic stellate cell proliferation and activation by glutamine metabolism

**DOI:** 10.1371/journal.pone.0182679

**Published:** 2017-08-10

**Authors:** Jiang Li, Mohammed Ghazwani, Ke Liu, Yixian Huang, Na Chang, Jie Fan, Fengtian He, Liying Li, Shizhong Bu, Wen Xie, Xiaochao Ma, Song Li

**Affiliations:** 1 Center for Pharmacogenetics, Department of Pharmaceutical Sciences, School of Pharmacy, University of Pittsburgh, Pittsburgh, PA, United States of America; 2 Department of Cell Biology,Municipal Laboratory for Liver Protection and Regulation of Regeneration, Capital Medical University, Beijing, China; 3 Department of Surgery, School of Medicine, University of Pittsburgh, Pittsburgh, PA, United States of America; 4 Department of Biochemistry and Molecular Biology (F.H.), College of Basic Medical Sciences, Third Military Medical University, Chongqing, China; 5 Diabetes Research Center, School of Medicine, Ningbo University, Ningbo, Zhejiang, China; University of Navarra School of Medicine and Center for Applied Medical Research (CIMA), SPAIN

## Abstract

Liver fibrosis is the excessive accumulation of extracellular matrix proteins, which is mainly caused by accumulation of activated hepatic stellate cells (HSCs). The mechanisms of activation and proliferation of HSCs, two key events after liver damage, have been studied for many years. Here we report a novel pathway to control HSCs by regulating glutamine metabolism. We demonstrated that the proliferation of HSCs is critically dependent on glutamine that is used to generate α-ketoglutarate (α-KG) and non-essential amino acid (NEAA). In addition, both culture- and in vivo-activated HSCs have increased glutamine utilization and increased expression of genes related to glutamine metabolism, including GLS (glutaminase), aspartate transaminase (GOT1) and glutamate dehydrogenase (GLUD1). Inhibition of these enzymes, as well as glutamine depletion, had a significant inhibitory effect on HSCs activation. In addition to providing energy expenditure, conversion of glutamine to proline is enhanced. The pool of free proline may also be increased via downregulation of POX expression. Hedgehog signaling plays an important role in the regulation of glutamine metabolism, as well as TGF-β1, c-Myc, and Ras signalings, via transcriptional upregulation and repression of key metabolic enzymes in this pathway. Finally, changes in glutamine metabolism were also found in mouse liver tissue following CCl4-induced acute injury. Conclusion: Glutamine metabolism plays an important role in regulating the proliferation and activation of HSCs. Strategies that are targeted at glutamine metabolism may represent a novel therapeutic approach to the treatment of liver fibrosis.

## Introduction

Liver fibrosis is the result of chronic liver damage such as chronic HCV infection, alcohol abuse, and nonalcoholic steatohepatitis (NASH), which is characterized as an excessive accumulation of extracellular matrix (ECM) [1–3]. It is considered as a model of the wound-healing response to chronic liver damage. With the persistent liver fibrosis, liver architecture is distorted and liver function is compromised afterwards, which results in hepatic insufficiency and portal hypertension, respectively. It can eventually lead to cirrhosis and hepatocellular carcinoma [1]. Enormous studies have been conducted to investigate the mechanism of liver fibrosis development [2, 4–8]. Hepatic stellate cells (HSCs) have received a lot of attention for the last few decades. HSCs were identified as the main collagen-producing cells in the liver after going through a sophisticated process of transactivation or transdifferentiation and becoming myofibroblast-like cells [9]. These activated HSCs acquire the ability to grow rapidly and produce large amounts of collagens, which are the major components of ECM [10, 11]. Several signal pathways have been identified that play important roles in modulating the functions of HSCs. Nonetheless, the mechanisms of activation of HSCs are not fully elucidated.

Glutamine (GLN), one of the nonessential amino acids, has important and unique metabolic functions. It is a precursor for the synthesis of other amino acids, proteins, nucleotides, and many other biologically important molecules, as being the most abundant free amino acid in the circulation and in intracellular pools [12–14]. GLN can be converted to α-ketoglutarate (α-KG), which provides carbon to TCA cycle, or converted to other NEAA by transaminases (GOT1 & GOT2) [15, 16]. GLN can also be converted to glutamate and pyrroline-5-carboxylate (P5C), which can stimulate collagen biosynthesis in cultured cells [17]. Reduction of P5C to proline is a critical step for proline biosynthesis, which has enormous effect on collagen synthesis as proline and hydroxyproline together comprise approximately 23% of the collagen molecules [18–20]. Recently, a few types of cancer cells have been shown to have addiction to increased GLN metabolism to fuel anabolic processes. This metabolism reprogramming is essential to maintain rapid cell proliferation [15, 21]. Drugs that are targeted at GLN metabolism are currently being examined as a new therapy for the treatment of cancers [22]. CB-839, a potent GLS inhibitor, was well tolerated and demonstrated excellent antitumor activity in preclinical studies [23].

It has been reported that transdifferentiation of quiescent HSC into myofibroblasts required increased glycolysis which led to lactate accumulation [24]. Glucose and glutamine metabolisms are interrelated, as both are precursors in the TCA cycle to generate energy, as well as precursors in the production of lipids, nucleotides, and amino acids [24]. However, the effect of glutamine metabolism on HSCs has not been studied. Here, we report that glutamine is essential to the proliferation of HSCs. Activated HSCs have greater glutamine metabolism rate. Several important signaling pathways contribute to the regulation of glutamine metabolism in HSCs.

## Materials and methods

### Reagents and chemicals

Carbon tetra-chloride (CCl_4_) was purchased from Merck (Whitehouse Station, NJ). Dulbecco's Modified Eagle Medium (DMEM) with and without L-glutamine, α-KG and NEAA were purchased from Life Technologies (Grand Island, NY). Bptes, EGCG, AOAA, 2-DG, 10058-F4, TGF-β1, GDC-0449 and 5-bromo-2'-deoxyuridine (BrdU) were purchased from Sigma-Aldrich (MO, USA). Collagen 1A1 antibody and BrdU antibody were purchased from Santa Cruz Biotech (Dallas, Texas).

### Animal care

Male, 6–8 weeks old mice and retired breed rats were housed and maintained in specific pathogen-free conditions in a facility approved by the American Association for Accreditation of Laboratory Animal Care under National Institutes of Health Guidelines. Food and water were provided ad libitum to the animals in standard cages. All experiments were performed in accordance with the guidelines of the Institutional Animal Care and Use Committee at the University of Pittsburgh. Mice were sacrificed using CO2 followed by cervical dislocation method.

### Cell line

LX2, an immortalized human hepatic stellate cell line, was kindly provided by Dr. Scott L. Friedman (Mount Sinai School of Medicine, New York, NY) and cultured in Dulbecco’s modified Eagle’s medium (DMEM) with 10% fetal bovine serum (FBS) and antibiotics. They were treated with the adipogenic differentiation mixture (MDI, 0.5 mM isobutylmethylxanthine, 1μM dexamethasone, and 1μM insulin) and incubated for 24 h.

### Rat and human HSC isolation

Male Sprague-Dawley rats (200–250 g) from Charles River Laboratories (Wilmington, MA) were used for rat HSC isolation. Human Non-Parenchymal Cells (NPCs) obtained from the donor livers were used for human HSC isolation. HSCs were isolated via in situ proteinase/collagenase perfusion followed by density gradient centrifugation as described [8]. The purity of isolated cells was >90%. Isolated HSCs were cultured in 6-well plates in DMEM with 10% FBS and antibiotics for 7 days to allow the process of activation. All animal work was approved by Institutional Animal Care and Use Committee (IACUC) of University of Pittsburgh and in accordance with the approved guidelines.

### Human specimens

Normal human liver samples were collected from 5 patients undergoing hepatic resection for colorectal metastasis (n = 5). Fibrotic samples (fibrosis stage: F2-4) were obtained from 16 livers of patients undergoing liver transplantation. Fibrosis was consecutive to chronic hepatitis C virus (n = 4) or hepatitis B virus (n = 10) infections, and alcohol-induced liver disease (n = 2). All tissues were obtained with donor consent by writing and the approval of the Capital Medical University Ethics Committee (approval number: 2011SY08).

### Real-time polymerase chain reaction

Total RNA was extracted with TRIzol reagent (Invitrogen). The total RNA was measured by NanoDrop 2000 (Thermo Scientific). Extracted RNA concentration was adjusted to be 1 micro gram per reverse transcription reaction using SuperScript III reverse transcriptase (Invitrogen). The primers for GLUD1, col1A1, actin2, PPAR-γ, GOT1, GOT2, GLS, GAPDH and ACTB were obtained from MWG Biotech. After synthesis of first strand cDNA, real-time PCR was performed using SYBR Green-based assays with the ABI Prism 7300 (Applied Biosystems, Foster City, CA) [8].

### Fluorescence microscopy for detection of autofluorescence of vitamin A

To investigate the effects of glutamine metabolism on the HSC activation, primary HSCs were treated with glutamine-deficient medium for 7 days. The cells were fixed in 4% paraformaldehyde and examined under a fluorescent microscope and photographed for documentation.

### BrdU staining, cell counting

LX2 cells or primary HSCs were seeded in 96-well plates and incubated in DMEM containing 10% FBS overnight. Cells were then treated with various concentrations of drugs (in DMSO) for 24–72 hrs. BrdU labeling and immunostaining analysis were performed as described [25], using an antibody for BrdU (Accurate; diluted 1:10,000). Cell counting was done by using a counting chamber. Briefly, cells were trypsinized first and then re-suspended in medium. 1μl was added to the chamber and cell numbers were counted under a microscope.

### Immunofluorescence

Primary HSCs were grown on chamber slides and then treated with various concentrations of drugs (in DMSO). Thirty-six hrs following the treatment, cells were fixed in 4% paraformaldehyde/PBS for 15 min at room temperature and washed with PBS three times. Cells were blocked with 2% bovine serum albumin in PBS for 1 h followed by incubation with anti-col1A1 antibody (Santa Cruz Biotechnology) (1:200) for 16 h at 4°C. After washing with PBS, a rhodamine-labeled secondary antibody (1:1000) was applied and incubated for 60 min. After an additional washing, cells were mounted and analyzed by fluorescence microscopy.

### LS-MS analysis of amino acid

#### Derivatization

Cell extracts (methanol/water = 50/50, v/v) were mixed with stable isotope labeled internal standards (d7-proline) and the mixture was lyophilized at -20°C in autosampler vials. The dry extracts were derivatized with 50 μL of a 10 mg/mL methoxyamine hydrochloride solution in pyridine for 30 min at 40°C. Subsequently, the extracts were silylated for 60 min at 80°C with 50 μL MTBSTFA (with 1% t-BDMCS).

#### GC-QTOF analysis

The derivatized samples were analyzed with an Agilent 7890A GC system (Agilent, Santa Clara, CA, USA) coupled to a quadrupole TOF mass spectrometer, G2-S QTOF (Waters Corporation, Manchester, UK), operating in APGC mode. The GC separation was performed using a fused silica DB5-MS capillary column (30m x 250μm I.D., 0.25μm film thickness; J&W Scientific, Folson, CA, USA). The initial GC oven temperature was 70°C. One min after injection, the GC oven temperature was increased with 15°C/min to 320°C and held for 4 min at 320°C. Splitless injections of 1 μL using a straight empty deactivated liner from Restek were carried out at 240°C. Helium was used as carrier gas at 2.0 mL/min. The interface temperature was set to 250°C using N_2_ as auxiliary gas at 400 L/h, and cone gas at 150 L/h. The APCI corona pin was operated at 3.0 μA. The APGC source was operated in proton transfer mode by placing an uncapped vial with water (modifier) in a specially designed holder located in the source door (REF). The QTOF detection was operated in full-scan mode (m/z 50–650) with the resolution at ~20,000.

### Statistical analysis

All data are expressed as mean (SD) unless otherwise stated. Comparisons between two groups were made with unpaired Student's t test. Comparisons among three or more groups were made with analysis of variance followed by Tukey-Kramer post hoc analysis. In all cases, P < 0.05 was considered statistically significant.

## Results

### 1: Glutamine is essential for the proliferation of HSCs

**Fig 1:**
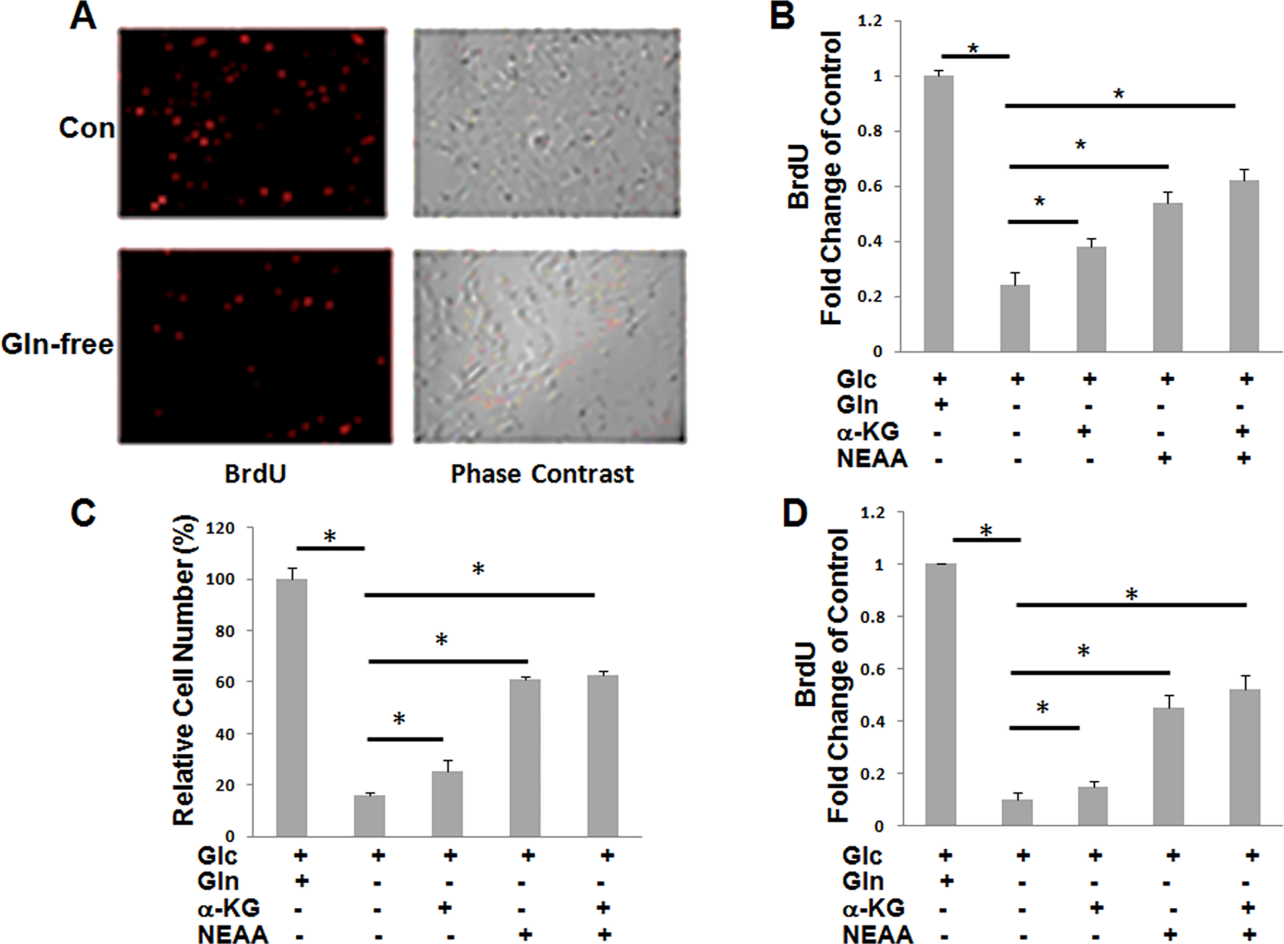
Glutamine is essential for HSCs proliferation. A&B: relative proliferation of LX2 cells (A,B) or primary HSCs (D) by BrdU staining or cell counting (C). Cells were plated in complete medium or Gln deficient medium with or without α-KG, NEAA, or the mixture of the two. BrdU-positive cells were quantified per optical section. Relative BrdU-positive cells for each group were analyzed. Error bars represent s.d. of triplicate samples from a representative experiment. *P < 0.05.

LX2 cells, an established immortalized human hepatic stellate cell line, were initially used to examine the effect of glutamine on cell proliferation. In the absence of glutamine, cell proliferation was markedly reduced as examined by BrdU assay (Fig 1A). Since α-KG and nonessential amino acids (NEAA) are the two of the major metabolites of glutamine, we then examined whether the inhibition of proliferation can be rescued via adding α-KG, NEAA or the mixture of the two. Cell proliferation was partially restored via the addition of either α-KG or NEAA, with NEAA being more effective than α-KG. The effect of NEAA alone was similar to that of the mixture of NEAA and α-KG (Fig 1B). Similar results were obtained when the effect of glutamine depletion was examined by cell counting (Fig 1C). We further examined the effect of glutamine depletion on the proliferation of primary HSCs via BrdU assay. HSCs were isolated from rats and cultured for 7 days to allow transactivation prior to exposure to glutamine-depleted medium. As shown in Fig 1D, glutamine depletion showed an effect on primary HSCs that was similar to that on HSC cell line (Fig 1A & 1B).

### 2: Inhibition of glutamine metabolism in HSCs has negative effect on cell proliferation

**Fig 2:**
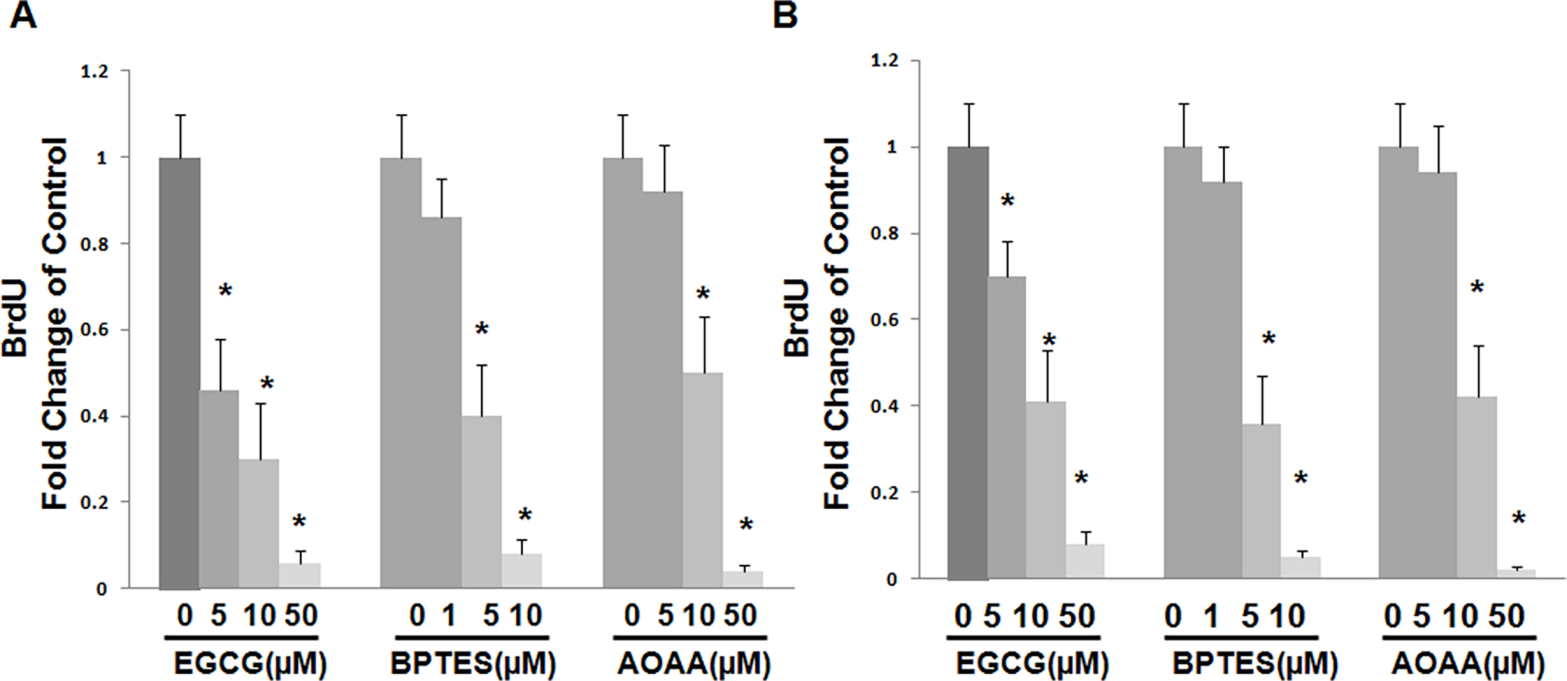
Gln metabolism enzyme inhibitors suppress HSCs proliferation. A&B, relative proliferation of LX2 cells (A) or primary HSCs (B) by BrdU staining assay. Cells were plated in complete medium and treated with Bptes (GLS inhibitor), EGCG (GLUD1 inhibitor) or AOAA (transaminase inhibitor) at different concentrations for 72 hrs.

The above data suggest that glutamine metabolism plays an important role in the proliferation of HSCs. To further confirm the role of glutamine metabolism in HSCs, we examined the inhibitory effects of glutaminase (GLS) inhibitor, Bptes, GLUD1 inhibitor, EGCG, and a pan inhibitor of transaminases inhibitor, AOAA on cell proliferation. GLS is involved in the 1^st^ step of glutamine metabolism via catalyzing the conversion of glutamine to glutamate while GLUD1 catalyzes the conversion of glutamate to α-KG. Transaminases are involved in the conversion of glutamate to α-KG and NEAA. As shown in Fig 2A, all of the three inhibitors inhibit the proliferation of LX2 cells in a concentration-dependent manner. Similar results were obtained in primary rat HSCs (Fig 2B). GLS and GOT1 siRNA were transfected in LX2 cells to silence GLS and GOT1. LX2 cells with GLS or GOT1 knockdown showed significant reduced cell proliferation (S1 Fig), which is consistant with study of inhibitors.

### 3: Glutamine metabolism is increased during HSCs transactivation

**Fig 3:**
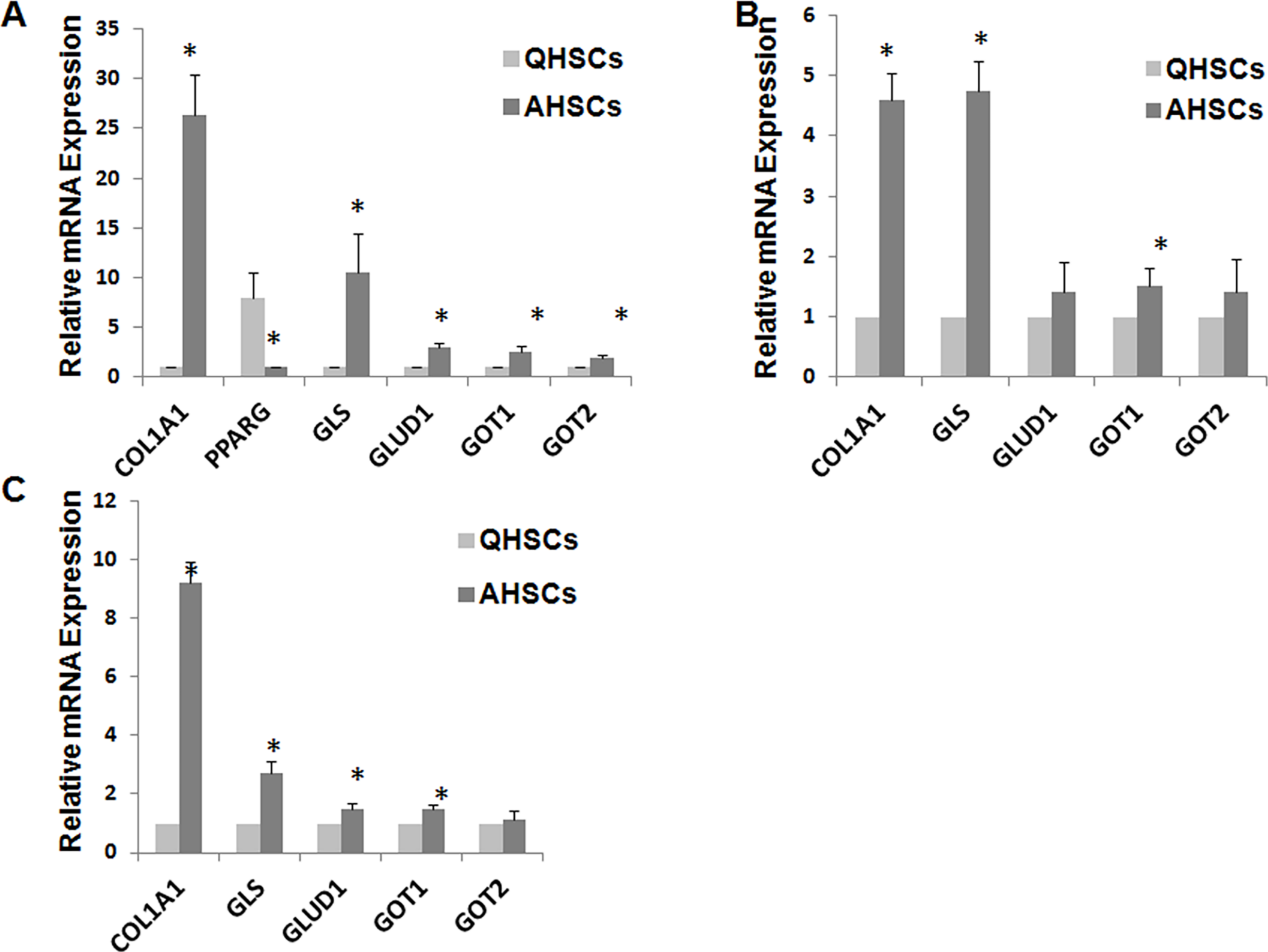
Gln metabolism is reprogrammed during HSC transactivation. A, primary HSCs isolated from rats were cultured for 7 days. B, primary HSCs isolated from donor patients were cultured for 7 days. C, HSCs were isolated from control mice and mice with liver fibrosis induced by 8 weeks of CCl_4_ treatment. Relative changes of mRNA expression of Gln metabolism genes were examined by RT-PCR.

The above data clearly suggest that glutamine is essential for the proliferation of HSCs. It is known that activated HSCs are highly proliferating compared to quiescent HSCs. Therefore, we hypothesized that glutamine metabolism is significantly increased during the transactivation of HSCs. Indeed, a preliminary study showed that the uptake of glutamine was significantly increased during the transactivation of primary rat HSCs (data not shown). We then examined and compared the mRNA expression levels of GLS, GLUD1, GOT1 and GOT2 in quiescent and activated rat HSCs. HSCs were isolated from rats and cultured for 7 days to allow transactivation. Transactivation of HSCs was confirmed by increased mRNA expression levels of col1A1 and actin2, and decreased mRNA expression level of PPARg (Fig 3A). It is apparent that the mRNA expression level of GLS was drastically increased in transactivated HSCs compared to quiescent HSCs (Fig 3A). Transactivation of HSCs was also associated with increased mRNA expression levels of GLUD1, GOT1, and GOT2. The similar changes in glutamine metabolism were also found in human HSCs (Fig 3B).

Following the demonstration of increased glutamine metabolism in culture-activated HSCs, we went on to study whether increased glutamine metabolism also happens in *in vivo*-activated HSCs. Mice were treated with CCl_4_ for 8 weeks and HSCs were then isolated from these mice and compared to HSCs isolated from naïve mice with respect to the mRNA expression levels of several glutamine metabolizing genes. As shown in Fig 3C, HSCs isolated from CCl_4_-treated mice were fully activated as evident from significantly increased mRNA expression levels of col1A1 and actin2. These cells also showed significantly enhanced expression of GLS, GLUD1, and GOT1 compared to HSCs isolated from naïve mice. No significant difference was found between control HSCs and *in vivo*-activated HSCs in the expression level of GOT2.

### 4: Glutamine metabolism plays an important role in regulating transactivation of HSCs

**Fig 4:**
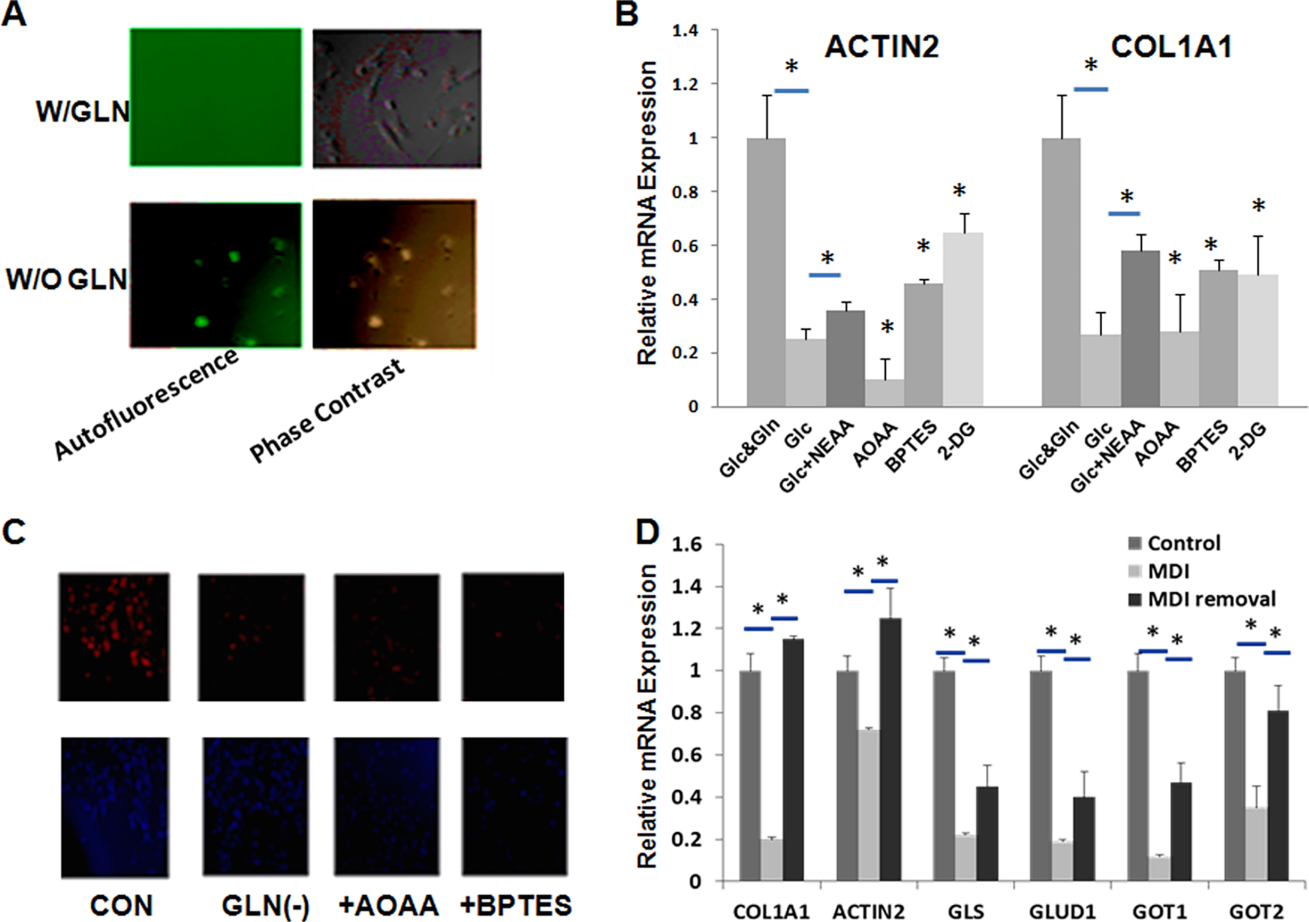
Gln metabolism influences transactivation of HSCs. A, primary HSCs were cultured for 7 days with or without glutamine. Autofluorescence was assessed. B &C, primary HSCs were cultured with or without Gln, Bptes, or AOAA. D, LX2 cells were treated with MDI. Relative changes of mRNA expression of Gln metabolism genes were analyzed by real-time PCR. Immunocytochemistry was used to assess the expression of col1A1 at protein level.

Given the importance of glutamine in HSCs proliferation and the dramatic changes in glutamine metabolism during transdifferentiation, we next examined whether glutamine plays any role in the transdifferentiation. Fig 4A shows that primary rat HSCs cultured on day 7 following isolation showed a typical myofibroblast morphology in normal medium. In contrast, HSCs cultured in glutamine-deficient medium retained their *quiescent* rounded *morphology*. In addition, these cells showed the characteristic *autofluorescence* of vitamin A storing *quiescent* HSCs (Fig 4A). Glutamine deficiency also had a dramatic inhibitory effect on primary cell proliferation (S2 Fig). With GLS or GOT1 knockdown, primary HSCs activation was inhibited by showing reduced expression of COL1A1 and ACTIN2 compared with control group (S3 Fig).

Fig 4B shows that the mRNA expression levels of actin2 and col1A1 were significantly downregulated in HSCs cultured in glutamine-deficient medium. Such downregulation was partially recused by supplementation with NEAA. Specific inhibitors of GLS and GOT showed a similar effect in downregulating the mRNA expression of actin2 and col1A1. As reported in literature [24], 2-DG, a glycolytic inhibitor, also showed significant inhibitory effect on cell transactivation.

Fig 4C shows the results of immunostaining of col1A1 in cells receiving different treatments. The expression of col1A1 was also significantly downregulated at protein level in rat HSCs that were cultured in glutamine-deficient medium or received treatment with AOAA or Bptes.

Adipogenic differentiation mixture (MDI) has been shown to induce the phenotypic reversal of activated HSCs to quiescent HSCs [26]. We then examined if such event is associated with phenotypic reversal in glutamine metabolism. LX-2 cells were treated with MDI medium for 24 h and then subjected to analysis of the mRNA expression levels of several glutamine metabolizing genes. As reported in literature, MDI treatment led to decreased mRNA expression levels of both col1A1 and actin2, suggesting successful “reversal” to “quiescent” HSCs (Fig 4D). It is also apparent that MDI treatment resulted in downregulation of GLS, GLUD1, GOT1 and GOT2. These effects of MDI were significantly attenuated when the cells were put back to normal medium for 24 h.

### 5. Glutamine-proline metabolic pathway is enhanced in activated stellate cells

**Fig 5:**
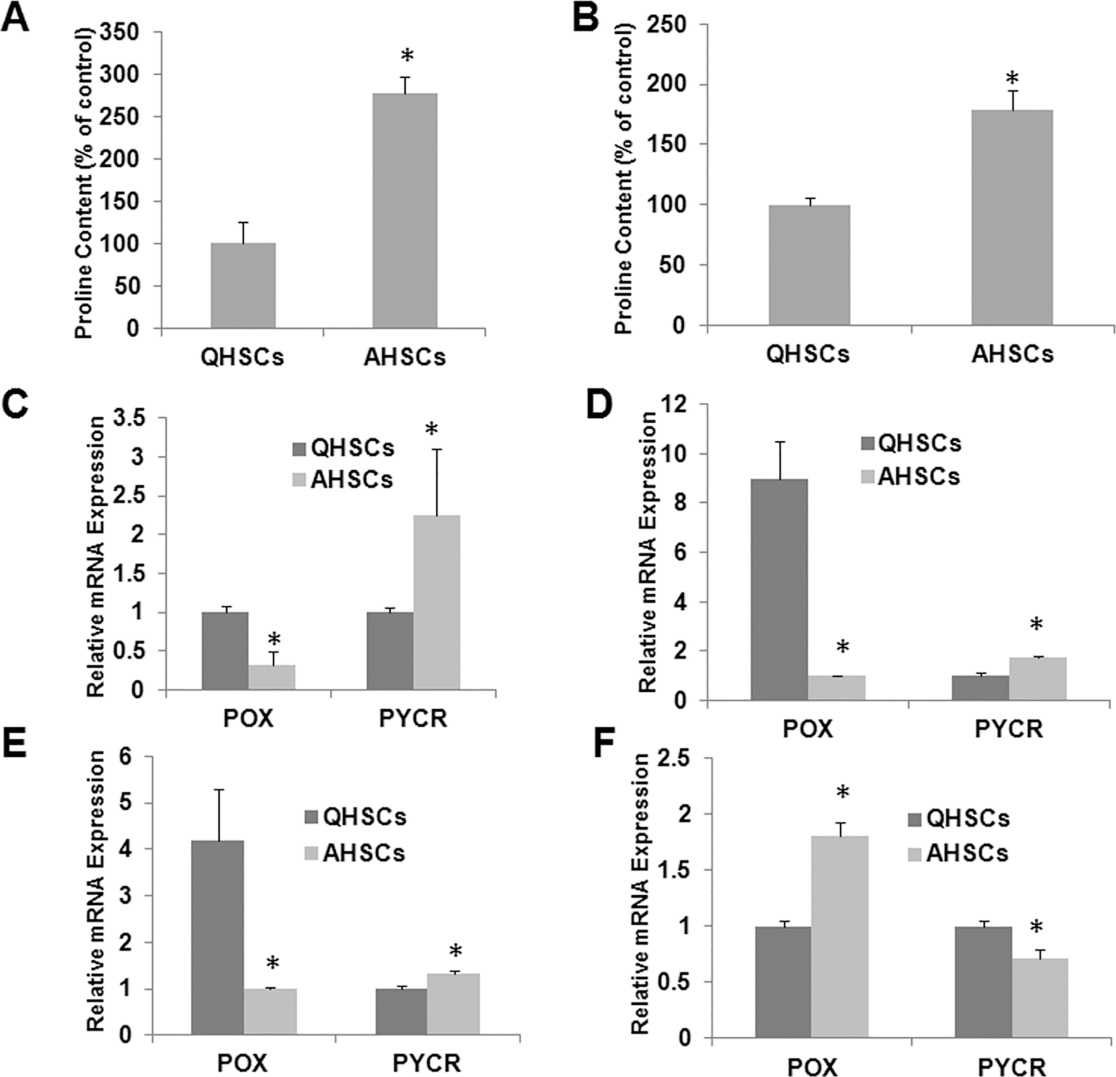
Gln-Pro metabolism is reprogrammed during HSC transactivation. A & C, rat primary HSCs were cultured for 7 days. B & E, primary HSCs were isolated from control mice and mice with liver fibrosis induced by 8 weeks of CCl_4_ treatment. D, human primary HSCs were cultured for 7 days. F, HSCs were treated with MDI. Relative changes of mRNA expression of proline metabolizing genes were measured by RT-PCR (C-F). Proline abundance in cultured HSCs was measured by mass spectrometry (A & B).

In addition to highly proliferating phenotype, activated HSCs are highly fibrogenic, producing large amounts of extracellular matrix proteins such as collagen. Proline is abundant in collagen and is important in the formation of the triple-stranded helix of collagen. We hypothesized that proline pool is significantly increased in activated HSCs. Fig 5A shows the result of MS analysis of free proline in quiescent and activated rat HSCs. It is apparent that the proline pool in HSCs was significantly increased during transactivation.

Intracellular proline may come from various sources including conversion from glutamine. To examine whether the increased proline pool in activated HSCs is due to an enhanced glutamine-proline metabolic pathway, deuterated glutamine was added to both quiescent and activated HSCs and the amount of deuterated proline metabolite was determined by mass spectrometry 24 h later. As shown in Fig 5B, there was significantly greater amount of deuterated proline in activated HSCs compared to that in quiescent HSCs.

We then examined the mRNA expression levels of PYCR and POX, the two enzymes that are involved in the interconversion between glutamine and proline. As shown in Fig 5C, there is a significant increase in the mRNA expression level of PYCR in culture-activated rat HSCs compared to quiescent HSCs. In contrast, the mRNA expression level of POX was drastically decreased in culture-activated rat HSCs. Similar results were found in culture-activated HSCs (Fig 5D*) and in vivo*-activated mouse HSCs (Fig 5E).

We further examined the gene expression levels of PYCR and POX in LX-2 cells before and after treatment with MDI medium. As shown in Fig 5F, MDI treatment led to a decrease in the mRNA expression level in PYCR and a concomitant increase in the expression level of POX, a result that was opposite to that seen in transactivation of HSCs (Fig 5C, 5D & 5E).

### 6: Hedgehog signaling regulates glutamine metabolism in HSCs, as well as TGF-β1, c-Myc and Ras signaling

**Fig 6:**
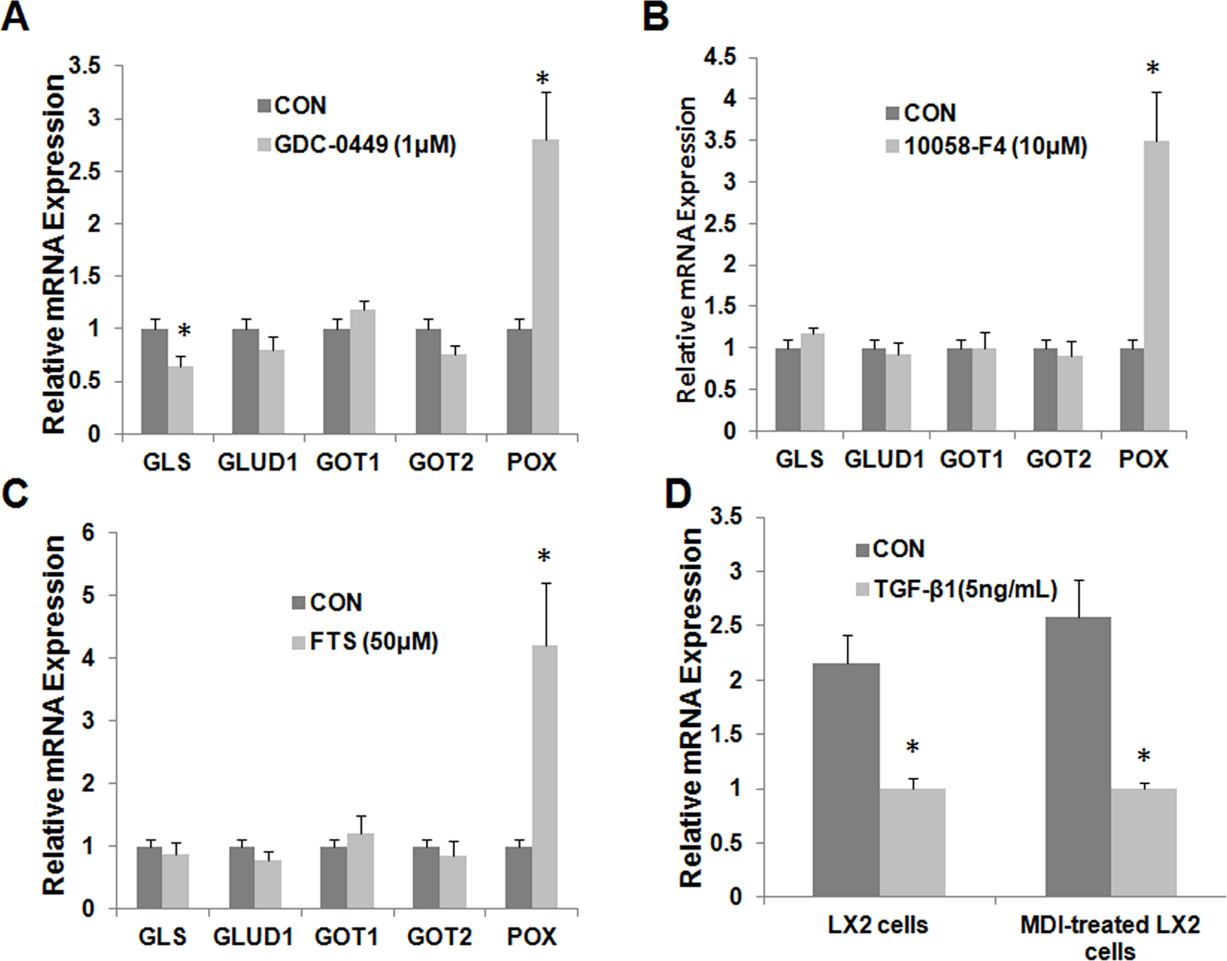
Gln metabolism is reprogrammed by Hedgehog signaling, Ras, Myc and TGF-β1. Primary HSCs were treated with GDC-0449 (to inhibit SMO) (A), FTS (B) (Ras inhibitor), 10058-F4 (C) (Myc inhibitor). LX2 cells and MDI-pretreated LX2 cells were treated with TGF-β1. RT-PCR was used to analyze expression of glutamine metabolic enzyme genes.

Since hedgehog pathway controls HSC fate by regulating glucose metabolism [23], the effect of hedgehog on glutamine metabolism was examined by using GDC-0449, a direct Smoothened antagonist. Inhibition of Smoothened in primary activated rat HSCs caused significant suppression of GLS expression, as well as dramatic upregulation of POX. GDC-0449 showed no effect on the expression of other genes examined including GLUD1, GOT1 and GOT2 (Fig 6A). Both c-Myc and Ras signalings have been shown to be important players in regulation of transactivation of HSCs [27, 28]. The two signalings have also been reported to be involved in glutamine metabolism in cancer cells [29, 30]. Therefore, their effect on glutamine metabolism in HSCs was also studied. Farnesylthiosalicylic acid (FTS, Salirasib), a Ras farnesylcysteine mimetic, selectively disrupts the association of chronically active Ras proteins with the plasma membrane, which results in the inhibition of Ras transformation [31]. 10058-F4 is a cell-permeable thiazolidinone that specificallly inhibits the c-Myc-Max interaction and prevents transactivation of c-Myc target gene expression [32]. Fig 6B & 6C show that inhibition of either c-Myc or Ras signaling led to significant upregulation of POX while it showed minimal effect on the expression of other glutamine metabolizing genes in primary activated rat HSCs.

We further examined the effect of TGF-β1 signaling on glutamine metabolism as TGF-β1 is the most important profibrogenic factor that induces HSCs activation and stimulates collagen production [33]. As shown in Fig 6D, TGF- β1 resulted in a significant decrease in the mRNA expression level of POX in LX-2 cells. A similar result was obtained in MDI-treated LX2 cells (Fig 6D). TGF-β1 had no effect on the mRNA expression of other glutamine metabolizing genes (data not shown).

### 7: Glutamine metabolism is altered during CCl_4_-induced acute liver injury and in human fibrotic livers

**Fig 7:**
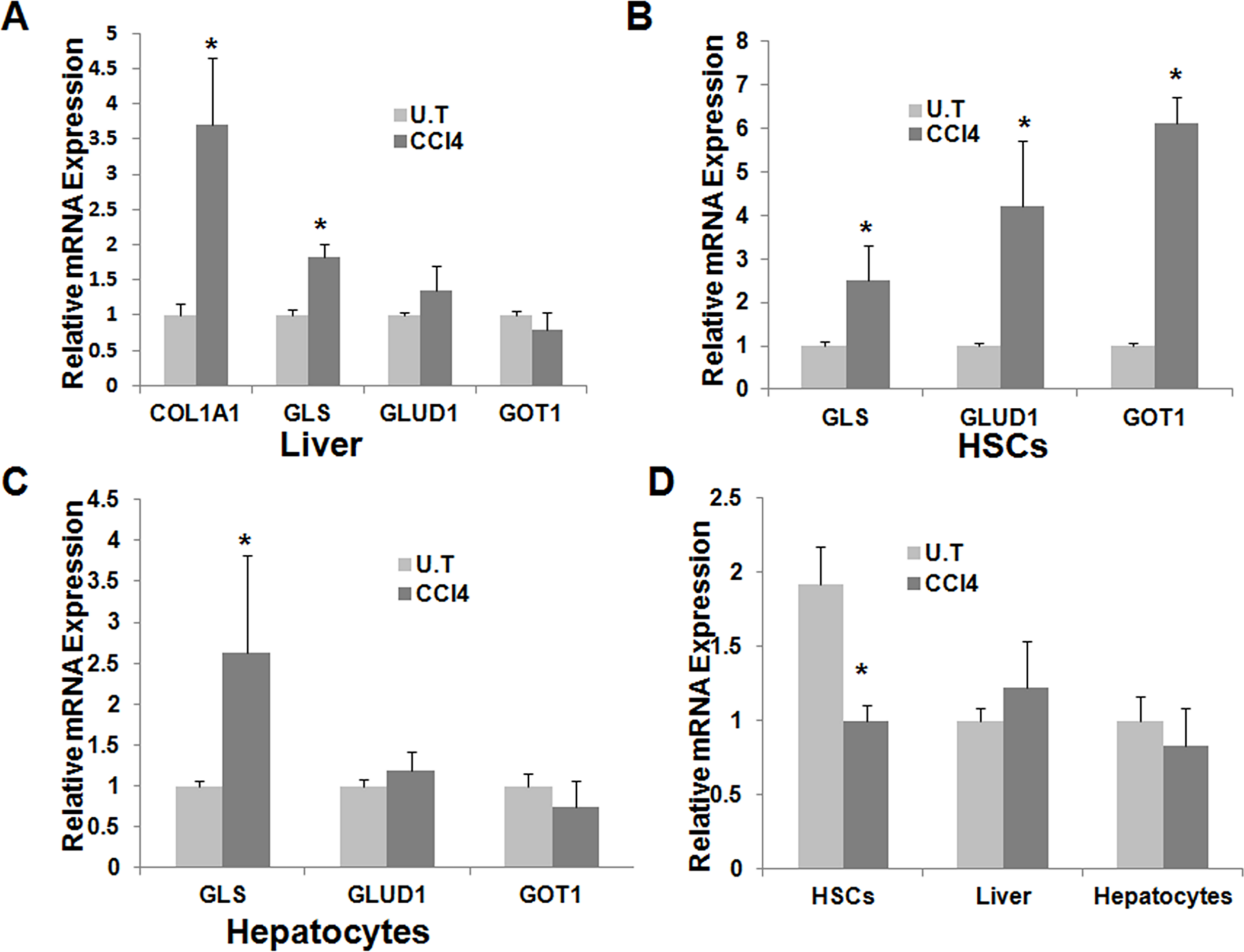
Gln metabolism is reprogrammed following acute liver injury. Mice received a single injection of CCl_4_ and the expression of several glutamine metabolizing genes in liver tissue (A), HSCs (B) or hepatocytes (C) were examined 2 days later. POX expression was also analyzed (D).

The above studies on glutamine metabolism were conducted with either culture-activated HSCs or the activated HSCs isolated from liver that was subjected to chronic injury (treatment with CCl_4_ for 8 weeks). To investigate whether glutamine metabolism is altered during the acute phase of liver injury, mice were treated with a single dose (100μl/kg) of i.p. CCl_4_ and liver was collected from the mice two days later. Changes in the mRNA expression levels of glutamine metabolizing genes were then examined in either liver tissue or the isolated cell populations. As reported in literature [3], acute CCl_4_ treatment led to a significant increase in the mRNA expression level of col1A1 in mouse liver (Fig 7A). We also saw significant upregulation of GLS mRNA expression in CCl_4_ -treated liver. No significant differences were found in the mRNA expression levels of GLUD1 and GOT1 between naïve mice and CCl_4_ -treated mice.

Fig 7B shows that the mRNA expression levels in isolated HSCs were significantly increased for GLS, GLUD1, and GOT1. These results were consistent with the data obtained from culture-activated HSCs or the activated HSCs isolated from mice exposed to chronic CCl_4_ treatment.

Fig 7C shows that the GLS mRNA expression was also significantly increased in the hepatocytes isolated from CCl_4_ -treated liver. No significant differences were found in the mRNA expression levels of other glutamine metabolizing genes between naïve mice and CCl_4_-treated mice.

We further examined the expression of POX following the CCl_4_ -induced acute liver injury. Fig 7D shows that the mRNA expression level of POX was significantly decreased in the HSCs isolated from CCl_4_ -treated mice in comparison with the HSCs isolated from naïve mice (Fig 7D). However, no significant differences were found between the two groups when the liver tissue or isolated hepatocytes were examined.

### 8: Glutamine metabolism is altered in human fibrotic livers

**Fig 8:**
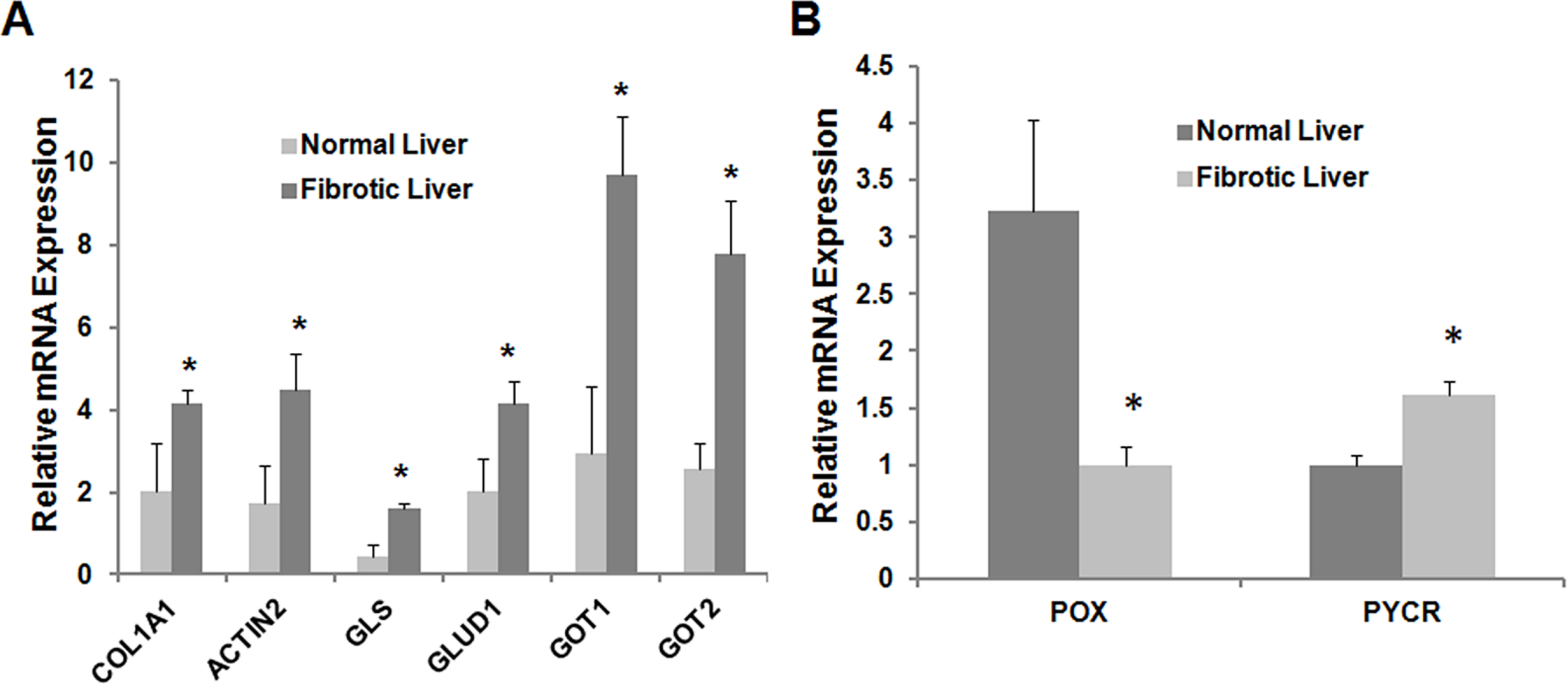
Gln-Pro metabolism is reprogrammed in human fibrotic livers. Liver tissues were collected from donor patients. RT-PCR was used to analyze expression of glutamine metabolic enzyme genes (A). Proline metabolizing genes were also analyzed (B).

The above data clearly suggested that altered glutamine metabolism is critically involved in both culture- and in vivo-activated HSCs. To establish their clinical relevance, we further examined the mRNA expression levels of several glutamine-metabolism genes in liver samples from patients with established liver fibrosis. As shown in Fig 8A, the expression levels of GLS, GLUD1, GOT1 and GOT2 were significantly increased in human fibrotic livers. Similar to what was observed in animal data, the expression level of POX was decreased in human fibrotic liver while that of PYCR was increased (Fig 8B).

## Discussion

Metabolic changes have long been identified to be an important event during cell change. Previously, these changes were believed to come after cellular changes. Now, more and more studies have proven that metabolic changes are key event for cell to make change or adjust to the environmental conditions. It has been well known that tumors display certain metabolic activities distinguishing them from normal tissues [34, 35]. The best-known metabolic abnormality in cancer cells is the Warburg effect, with an increased glycolysis even in the presence of oxygen [34]. Glutamine metabolic reprogramming also has been shown to be an important event of pancreatic cancer cells [36]. On the other hand, mutation of several metabolic genes such as IDH1, IDH2, SDH, and FH is sufficient to initiate tumors and lead to tumorigenesis. Moreover, certain metabolic changes have been indicated to contribute to T cell development, cell proliferation and death [37, 38]. In HSCs, the first report of metabolic changes during cell transactivation came from Dr. Diehl’s group (24). They found that there were significant changes to glucose metabolism in A-HSCs compared with Q-HSCs. In this study, we have shown for the 1^st^ time that glutamine metabolism is also significantly altered during the transactivation of HSCs.

Glutamine, the most abundant intracellular free amino acid, has many important functions. It can be converted to α-KG and enters TCA cycle as energy fuel or converted to other amino acids including proline [39–41]. Previous studies have shown that human diploid fibroblasts utilize both glucose and glutamine as energy sources. Skin fibroblast proliferation is dependent on glutamine supply [42, 43]. It is understandable that glutamine may serve as the major anaplerotic precursor in proliferating fibroblasts. During the transactivation process, HSCs change into rapid-growing, fibroblast-like type of cells. We hypothesized that there will be some significant changes occurred to meet the energy need and provide collagen synthesis material. Indeed, the significantly increased expression of GLS, GOT and GLUD1 was observed in in-vitro activated HSCs as well as in-vivo activated HSCs, which suggested an increased glutaminolysis. Measurement of intermediate of glutaminolysis also supported that A-HSCs have an increased glutamine metabolic rate. Any disruption of glutaminolysis pathway had enormous effect on cell proliferation and collagen production. Furthermore, inhibition of these changes can revert back the transactivation process. Glutamine synthetase(GS) has been reported to be induced during HSC activation in protein level. mRNA expression showed different patterns during the transactivation process. We haven’t observed significant changes of GS mRNA expression at day 1 and day 7 culture. Glutamine deficiency doesn’t affect the expression of GS. Increase of GS protein expression may contribute to regulation of glutamine metabolism in HSC, which may not be the dominant effect during the transactivation process. Thus, these data suggest that the increased glutamine metabolism does not simply represent an adaptive change to meet the increased energy demand. Rather, glutamine metabolism may be one of the important early players that are critically involved in the initiation of transactivation of HSCs.

The underlying mechanism for the altered glutamine metabolism during transactivation of HSCs is not completely understood at present. Hedgehog seems to have the most significant effect on regulation of glutamine metabolic changes. Hedgehog pathway has been studied and identified to be an important signaling pathway to control cell differentiation. Its activation can prepare cells to the damage [44]. For HSCs, Hh is activated during the transactivation process. It can stimulate Q-HSC to become A-HSC. One of the mechanisms is its effect on glycolysis, which leads to accumulation of lactic acid (24). Our studies suggest that the Hh-glutamine metabolism axis may be another important mechanism through which hedgehog signaling controls HSC activation. Hh inhibitor is the only agent among several pathway inhibitors tested that affected the expression of glutaminase, a key enzyme that is involved in the 1^st^ step of glutamine metabolism. Hh inhibitor also had significant impact on POX expression.

Ras is also activated in A-HSCs [28]. In some tumor cells, Ras is a major player of glutamine metabolism regulation [45–47]. In hepatic stellate cells, TGF-β1 induces activation of Ras, which may contribute to regulation of transdifferentiation and proliferation as well as stimulation of collagen synthesis by TGF-β1 [48]. In our study, the effect of Ras activation on glutamine metabolism is mainly through the regulation of POX. This effect may modulate the content of proline in HSCs. As indicated in a study from James J. Potter et al, a transient increase in c-Myc precedes the transdifferentiation of hepatic stellate cells [49]. c-Myc has been studied in several types of tumor cells showing enormous effect on regulation of cell metabolism. This effect has been proven to be one of major mechanisms for c-Myc-induced cell proliferation and cell transformation [50, 51]. In HSCs, c-Myc is activated in the very early stage of transactivation. With inhibition of Myc activation, the same effect of Ras inhibition has been observed on glutamine metabolism. TGF-β1, as a potent fibrogenic factor, also has a significant effect on proline metabolism through regulating POX expression based on our studies. Proline is abundant in collagen and is important in the formation of the triple-stranded helix of collagen. Any effect on proline will have dramatic effect on collagen synthesis. The fact that POX is regulated by several signalings also suggests its significance in HSC activation and the overproduction of collagen. It remains to be studied how the different signalings interact with each other in regulating the expression of POX.

Most of the noted changes in fully activated HSCs were well reproduced in a CCl_4_ -induced acute liver damage model. These data further support the notion that altered glutamine metabolism may be an important early event in regulating HSC transactivation and the subsequent fibrotic changes. It is interesting to note the expression level of glutaminase in isolated hepatocytes is also significantly increased following acute liver damage. The impact of altered glutamine metabolism on liver injury response remained to be investigated.

We have further shown that glutamine metabolism is altered in human fibrotic livers, recapitulating most of the findings observed in rodent (in vitro and in vivo) and isolated human HSCs. These studies suggest the clinical relevance of our findings. Taken together, there have been significant changes of glutamine metabolism occurred during in vitro and in vivo activation of HSCs (S4 Fig). Recently, GLS inhibitors are being examined as a new treatment for cancers. These compounds have been shown to be safe in both preclinical and clinical studies [22, 23] Strategies that are targeted at glutamine metabolism may also represent a novel therapeutic approach to the treatment of liver fibrosis.

## Supporting information

S1 Fig GLS and GOT1 silencing inhibit cell growth in LX2 cells. A&B, Cells were transfected with GLS or GOT1 siRNA for 3 days. Relative changes of mRNA expression of GLS or GOT1 were examined by RT-PCR. C: relative cell availability was analyzed by MTT assay.
(TIF)

S2 Fig Glutamine deficiency reduces cell availability in primary hepatic stellate cells. Primary cells were cultured in glutamine deficient medium for 7 days. Relative cell availability was analyzed by MTT assay.
(TIF)

S3 Fig GLS and GOT1 silencing affects HSCs transactivation. A&B, primary HSCs were transfected with GLS or GOT1 siRNA and cultured for 3 days. Relative changes of mRNA expression of GLS or GOT1 were examined by RT-PCR. C: primary HSCs were transfected with GLS or GOT1 siRNA and cultured for 7 days. Relative genes expression of ACTIN2 and COL1A1 were analyzed by RT-PCR.
(TIF)

S4 Fig A schematic diagram of changes of glutamine metabolism genes during HSCs activation. The schematic shows the various genes involved in the regulation of HSCs activation.
(TIF)
