# Supplementary figures and images for: Regulation of hepatic stellate cell proliferation and activation by glutamine metabolism
Source: PLoS One. 2017 Aug 10;12(8):e0182679. doi: 10.1371/journal.pone.0182679 (PMC5552314; doi:10.1371/journal.pone.0182679)

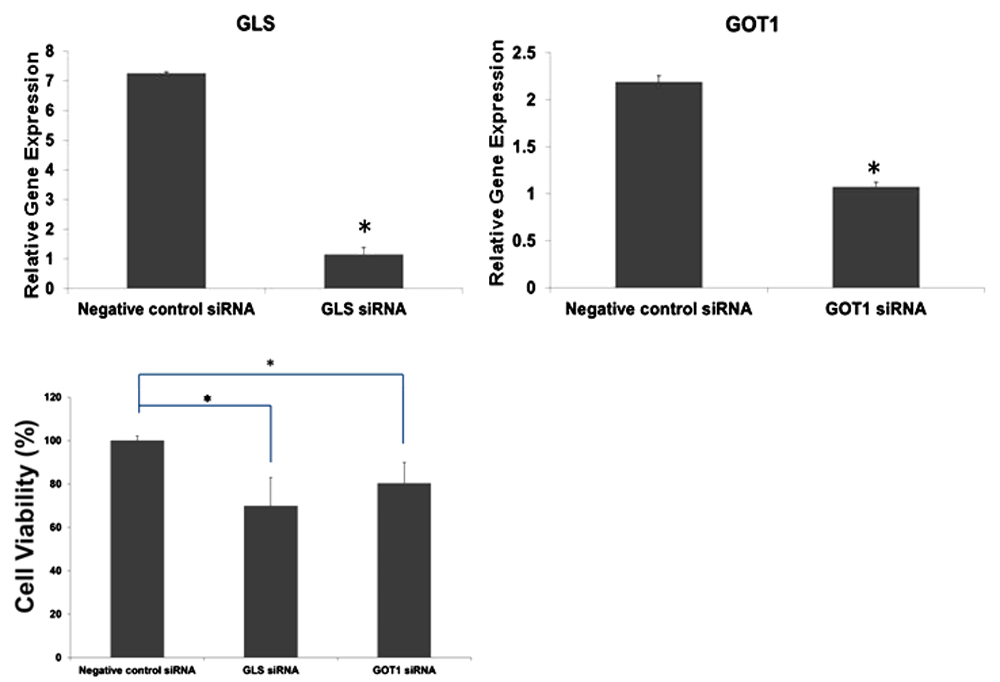

Supplement: S1 Fig — A&B, Cells were transfected with GLS or GOT1 siRNA for 3 days. Relative changes of mRNA expression of GLS or GOT1 were examined by RT-PCR. C: relative cell availability was analyzed by MTT assay. (TIF) [file pone.0182679.s001.tif]

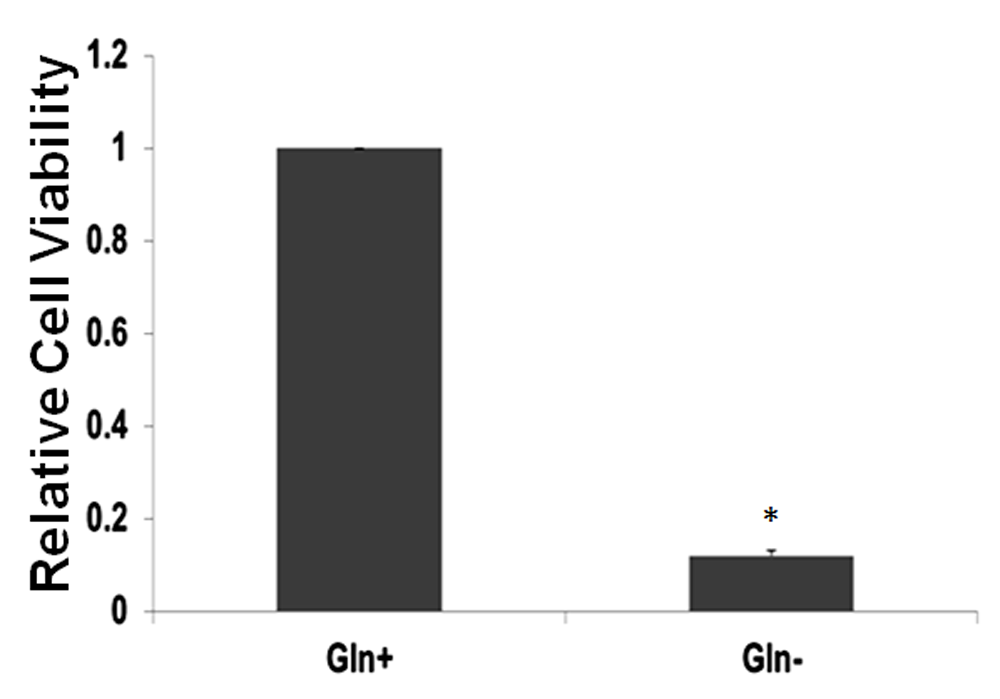

Supplement: S2 Fig — Primary cells were cultured in glutamine deficient medium for 7 days. Relative cell availability was analyzed by MTT assay. (TIF) [file pone.0182679.s002.tif]

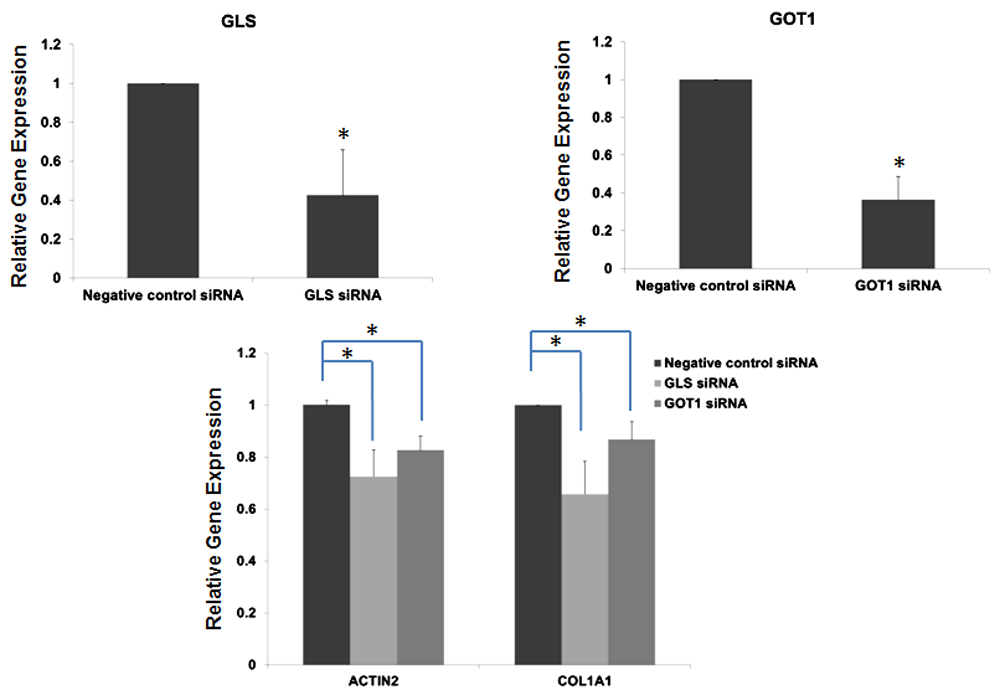

Supplement: S3 Fig — A&B, primary HSCs were transfected with GLS or GOT1 siRNA and cultured for 3 days. Relative changes of mRNA expression of GLS or GOT1 were examined by RT-PCR. C: primary HSCs were transfected with GLS or GOT1 siRNA and cultured for 7 days. Relative genes expression of ACTIN2 and COL1A1 were analyzed by RT-PCR. (TIF) [file pone.0182679.s003.tif]

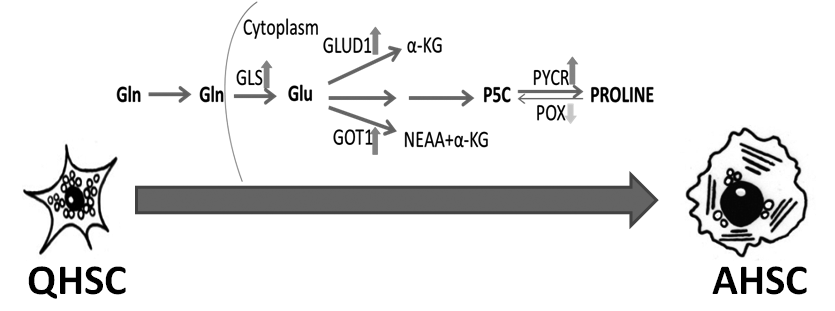

Supplement: S4 Fig — The schematic shows the various genes involved in the regulation of HSCs activation. (TIF) [file pone.0182679.s004.tif]
